# Supplementary material for: The interactome of intact mitochondria by cross-linking mass spectrometry provides evidence for coexisting respiratory supercomplexes
Source: Mol Cell Proteomics. 2017 Dec 8;17(2):216–32. doi: 10.1074/mcp.RA117.000470 (PMC5795388; doi:10.1074/mcp.RA117.000470)
Supplement: Supplemental Data [file supp_RA117.000470_133922_0_supp_23978_fzffwf.zip › spectra_annotation/mito_spectra_annotation/284-1-4-1-8-1.pdf]

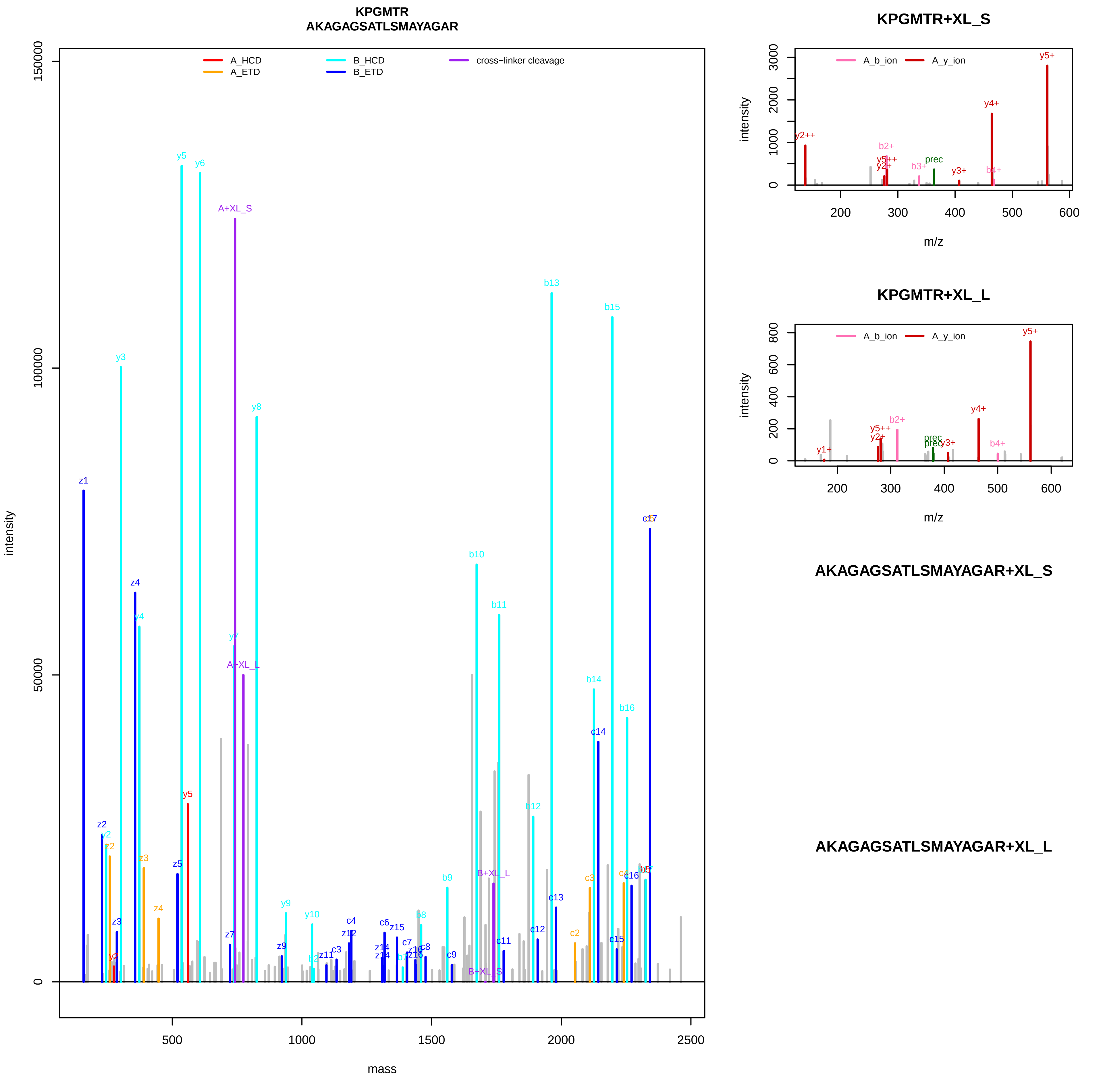

KPGMTR
AKAGAGSATLSMAYAGAR
A_HCD
A_ETD
B_HCD
B_ETD
cross−linker cleavage
intensity
mass
0
50000
100000
150000
500
1000
1500
2000
2500
z1
z2
y2
z2
z3
y2
y3
z4
y4
z3
z4
z5
y5
y5
y6
A+XL_S
A+XL_L
y7
z7
y8
z9
y9
y10
z11
c3
c4
z12
c6
z14
z14
z15
c7
c8
b8
b9
c9
b10
B+XL_L
B+XL_S
b11
c11
b12
c12
b13
c13
c2
c3
b14
c14
c15
b15
b16
c16
c17
KPGMTR+XL_S
A_b_ion
A_y_ion
intensity
m/z
0
1000
2000
3000
200
300
400
500
600
y2++
b2+
y5++
y2+
b3+
prec
y3+
y4+
b4+
y5+
KPGMTR+XL_L
A_b_ion
A_y_ion
intensity
m/z
0
200
400
600
800
200
300
400
500
600
y1+
y5++
y2+
b2+
prec
prec
y3+
y4+
b4+
y5+
AKAGAGSATLSMAYAGAR+XL_S
AKAGAGSATLSMAYAGAR+XL_L
